# Diagnostic Modalities in Heart Failure: A Narrative Review

**DOI:** 10.7759/cureus.67432

**Published:** 2024-08-21

**Authors:** Tanisha, Chaithanya Amudha, Mohammed Raake, Dany Samuel, Sulakshna Aggarwal, Zainab M Din Bashir, Karabo K Marole, Iqra Maryam, Zahra Nazir

**Affiliations:** 1 Department of Internal Medicine No. 4, O.O. Bogomolets National Medical University, Kyiv, UKR; 2 Department of Medicine and Surgery, Saveetha Medical College and Hospital, Chennai, IND; 3 Department of Surgery, Annamalai University, Chennai, IND; 4 Department of Radiology, Medical University of Varna, Varna, BGR; 5 Department of Neurology, Centre for Brain Research, Bengaluru, IND; 6 Department of Medicine and Surgery, Combined Military Hospital (CMH) Lahore Medical College and Institute of Dentistry, Lahore, PAK; 7 Department of Medicine and Surgery, St. George's University School of Medicine, St. George’s, GRD; 8 Department of Radiology, Allama Iqbal Medical College, Lahore, PAK; 9 Department of Internal Medicine, Combined Military Hospital, Quetta, PAK

**Keywords:** adm, gdf-15, galectin-3, ace inhibitors and angiotensin receptor blockers, heart failure

## Abstract

Heart failure (HF) can present acutely or progress over time. It can lead to morbidity and mortality affecting 6.5 million Americans over the age of 20. The HF type is described according to the ejection fraction classification, defined as the percentage of blood volume that exits the left ventricle after myocardial contraction, undergoing ejection into the circulation, also called stroke volume, and is proportional to the ejection fraction. Cardiac catheterization is an invasive procedure to evaluate coronary artery disease leading to HF. Several biomarkers are being studied that could lead to early detection of HF and better symptom management. Testing for various biomarkers in the patient's blood is instrumental in confirming the diagnosis and elucidating the etiology of HF. There are various biomarkers elevated in response to increased myocardial stress and volume overload, including B-type natriuretic peptide (BNP) and its N-terminal prohormone BNP. We explored online libraries such as PubMed, Google Scholar, and Cochrane to find relevant articles. Our narrative review aims to extensively shed light on diagnostic modalities and novel techniques for diagnosing HF.

## Introduction and background

One of the oldest definitions of heart failure (HF) by Wagner et al. [1] (1977) defines it as when either the systolic or diastolic operation of the ventricle is impaired to the degree that, despite compensatory mechanisms, the demands of the peripheral organs are not satisfied [1]. Over the years, the definition of HF has evolved into more nuanced and detailed clinical syndromes, with the American College of Cardiology/American Heart Association (ACC/AHA), Heart Failure Association (HFA)/European Society of Cardiology (ESC), and Japanese Heart Failure Society (JHFS) each having their spin on the topic [2-4]. Bozkurt et al. [2] broke down HF according to phenotype. They summarized that the definition of HF comes down to three points: evidence of structural heart disease, history of commonly reported HF symptoms, and objective signs commonly seen in HF.

In the 2019 HFA Atlas project, the median prevalence of HF across 13 European countries was estimated at 1.7%. The prevalence ranged from ≤1.2% in Greece and Spain to >3.0% per 100 persons in Lithuania and Germany [5]. The HF prevalence was 2.4% in the USA in 2012, expected to climb to 3.0% by 2030 [6], while among Canadians aged ≥40 years in 2012/2013, the age-standardized HF prevalence approximated 4% in males and 3% in females, according to the Public Health Agency of Canada [7]. Looking at the global picture, Savarese et al. state that the prevalence of HF ranges from 1% to 3% in adult population [8]. A UK study by Hancock et al. evaluated the barriers to accurate diagnosis of heart over 10 years, and lack of availability or knowledge of echocardiography was among them [9]. Another study by Smeets et al. highlighted doubts about the value and lack of confidence in the interpretation of results, as well as unawareness of the importance of HF classification led to difficulties in correctly diagnosing HF [10].

This narrative review aims to explore and evaluate various diagnostic modalities used in assessing HF, providing insights into their clinical utility and how they can be better integrated into the treatment of HF seamlessly.

## Review

Pathophysiology of heart failure

Understanding HF pathology is pivotal in exploring diagnostic modalities [11]. The heart's chambers are lined with muscles organized into sulci, also known as atrioventricular grooves, and the coronary sulcus, which contains essential structures. The heart is an organ almost entirely dependent on aerobic respiration, and its structure reflects this metabolic need [12-14].

The heart's muscle tissue is "involuntary" or "muscle tissue with no free ends," which regulates its automatic contraction. Gap junctions couple the entire population of ventricular myocardial cells and undergo excitability via organized electrical activity that regulates the functional morphology of the intact heart [14,15].

When the heart is at rest, the myocardial cells are also relaxed; following transmission of the electrical signals to induce cardiac muscle extrusion, the heart initiates a series of events, including ventricular contraction, aortic and ventricular valve opening, and blood expulsion [16]. The inability to contract leads to fluid accumulation in the lungs, resulting in symptoms such as shortness of breath and cough due to increased pressure in the pulmonary veins. Eventually, this leads to fluid accumulation in the veins throughout the body, causing swelling in the lower extremities and abdomen, enlargement of the liver, and sometimes fluid accumulation in the abdomen [17,18].

Several conditions, such as infections, systemic toxins, cardiotoxic drugs, and ischemic heart disease (IHD), precipitate HF by afflicting the heart's ability to pump; this occurs by damaging the myocardium, conductive system, and heart structure or a combination of the same. Numerous mechanisms bring about this condition [18]. Usually, the heart maintains a baseline volume of blood pumped over time. Stroke volume is the blood pumped by the ventricle in each heartbeat. Broadly, stroke volume depends on the heart's contractility, afterload, and preload [19].

Angiotensin II (ATII) can promote HF by generating growth, stimulating extracellular matrix protein production, reducing nitric oxide (NO) bioavailability, serous new formation, and causing hypertrophy. The activation of the renin-angiotensin-aldosterone system (RAAS) is one of the leading causes of the progression of the disease since ATII has a significant pathophysiological role in the progression of HF. It increases the afterload and leads to ventricular hypertrophy, myocardial oxygen consumption, cellular apoptosis, and necrosis, reducing the fraction or shortening of the ejection component, thus worsening ventricular remodeling. The arterial ventricular-ventricular reaction causes an increase in both. This leads to a further increase in the preload while reducing contractility [19].

Cytokines are a particular group of proteins or glycoproteins secreted by various cells as mediators of the immune response, which includes a wide variety of actions such as stimulation of the immune response and control of the development and activities of white blood cells and their precursors. Cytokines play a significant part in the development of both acute and chronic HF. Interleukin 1B (IL-1B), the primary mediator of the acute phase response, is increased after myocardial infarction or acute compression of the coronary arteries. IL-1B can induce the synthesis of cytokines through stimulation of other cells and thus initiate a cascade of proinflammatory responses. Interleukin 6 (IL-6) can act negatively on the myocardium through different pathways, binding mainly to the GP130 receptor in excess atria and ventricles. The induction of myocardial GP130 causes a direct negative inotropic effect and a hypertrophic response. Increased levels of IL-6 are found in ischemic, dilated, and hypertrophic cardiomyopathy and HF. Tumor necrosis factor-alpha (TNF-A) is synthesized from activated monocytes/macrophages; the increase in TNF-A in patients with HF has been related to disease severity and has a strong correlation with subsequent mortality [20].

Inflammation is, in many ways, a natural biological response. However, it can also be harmful and restorative. It is now generally accepted that inflammation is tightly associated with the initiation, evolution, and progression of a wide range of cardiovascular diseases, including myocardial infarction, stroke, atherosclerosis, diabetes, aging, and HF, that is, the end-stage of cardiovascular disease [21]. The continuous inflammation of the myocardium in the presence of chronic injury leads to several maladaptive responses, particularly changes in structure and functional impairment that collectively lead to HF. Notably, various cellular signaling pathways that regulate both immune and non-immune cell function have been shown to contribute to the risk of developing HF and determine the progression of the disease, thus emerging as attractive targets for HF therapy. Therefore, efforts to modulate inflammation may serve as a cornerstone for future interventions to treat and prevent HF [22]. Inflammatory pathways have been investigated as mediators and contributors to the processes affecting the myocardium and culminating in HF development. Both expression and increased circulating levels of classical inflammatory markers, including high-sensitivity C-reactive protein, intracellular adhesion molecule-1, and tumor necrosis factor-alpha, have been described in HF and associated with increased mortality, independent of traditional risk factors. Numerous genetic variants affecting immune system functionality and regulating gene expression of various proinflammatory genes, such as tumor necrosis factor-associated protein-A and the multiple variants of intracellular adhesion molecule-1, have been associated with the risk of developing HF. In terms of heart disease, the emergence of the principal risk factors and HF correlates with conditions of increased oxidative stress. Oxidative stress has increased primarily in the failing human heart, enhanced mitochondrial reactive oxygen species (ROS) generation, and reduced antioxidant defense. ROS may also negatively affect cardiac remodeling and function by inducing apoptosis, fibrosis, hypertrophy, or endothelial dysfunction. In failing hearts, both sources of ROS and their destroyed targets are stimulated, suggesting potential cascades of pathological events involving excessive ROS generation in human HF [23].

The HF types will be described according to the ejection fraction classification, defined as the percentage of the blood volume that exits the left ventricle after myocardial contraction, undergoing ejection into the circulation, also called stroke volume, and is proportional to the ejection fraction. The gold standard for obtaining this information is the cardiac catheter procedure, which allows for the most accurate ejection fraction assessment, calculates other cardiac function and structure parameters, identifies associated complications, such as ischemia, and allows the best therapeutic approach. HF can simultaneously affect either the contractile (systolic phase), the relaxatory phase (diastolic phase), or both. Patients may present with reduced ejection fraction (<40%). The patients may have preserved ejection fraction (>50%). Finally, if the ejection fraction is between 40% and 49%, it is considered that their ejection fraction is mid-range.

Systolic HF stems from excessive loading conditions. It consists of a significant impairment of ejection fraction despite normal to elevated left ventricular (LV) diastolic volume, predominantly regulated by the intrinsic myocardial contractile function. Overzealous ventricular remodeling and molecular cardiac stress markers, including B-type natriuretic peptide (BNP), N-terminal pro-B natriuretic peptide (NT-proBNP), cardiac troponin T (cTnT), and cardiac troponin I (cTnI), are predominant compared to diastolic HF. The risk for the determinant events of this cardiac scenario and end simulation includes LV hypertrophy to heart weight ratio, myocyte diameter, interstitial fibrosis coupled with hemodynamic overload, and high cTnT and cTnI plasma levels. Myocardial pathological structural changes are regulated by proinflammatory cytokines and macrophage-activating pro-fibrotic anti-inflammatory cytokines, the latter scaffold for ventricular myofibroblasts leading to extracellular matrix overproduction in which IL-10 and transforming growth factor beta (TGF-β) are significant components [24,25].

Acute decompensated HF is defined as a sudden structural change in the heart's ability to output, which leads to poor systemic perfusion at rest or with minimal activity [26].

IHD commonly causes congestive heart failure (CHF). Hyperuricemia is a particular type of chronic pathogenic mechanism of CHF, and it has been found to indirectly increase the risk of coronary disease events in recent years. Other important causes are hypertensive HF and valvular heart diseases, including both stenosis and regurgitation. It has become the leading cause of death in the general population [27].

There are a myriad of non-modifiable risk factors for developing HF. These include age, gender, race, and ethnicity. Modifiable lifestyle-related risk factors also include inactivity or sedentary behavior, poor diet, smoking, excessive alcohol consumption, and obesity. Other modifiable risk factors may involve high blood pressure, diabetes, and high cholesterol levels. A family history of HF is a significant independent predictor of the incidence of HF. Specifically, a history of heart disease in one's parents, and not a history of hypertension, is a significant predictor of HF. In the USA, the prevalence of HF, however, increases with age beginning at age 65. The lifetime risk of HF, however, is greater than 20% in both sexes. The natural turnover of myocardial tissue has been proposed as a mechanism of age-related cardiac aging [28].

**Figure 1:**
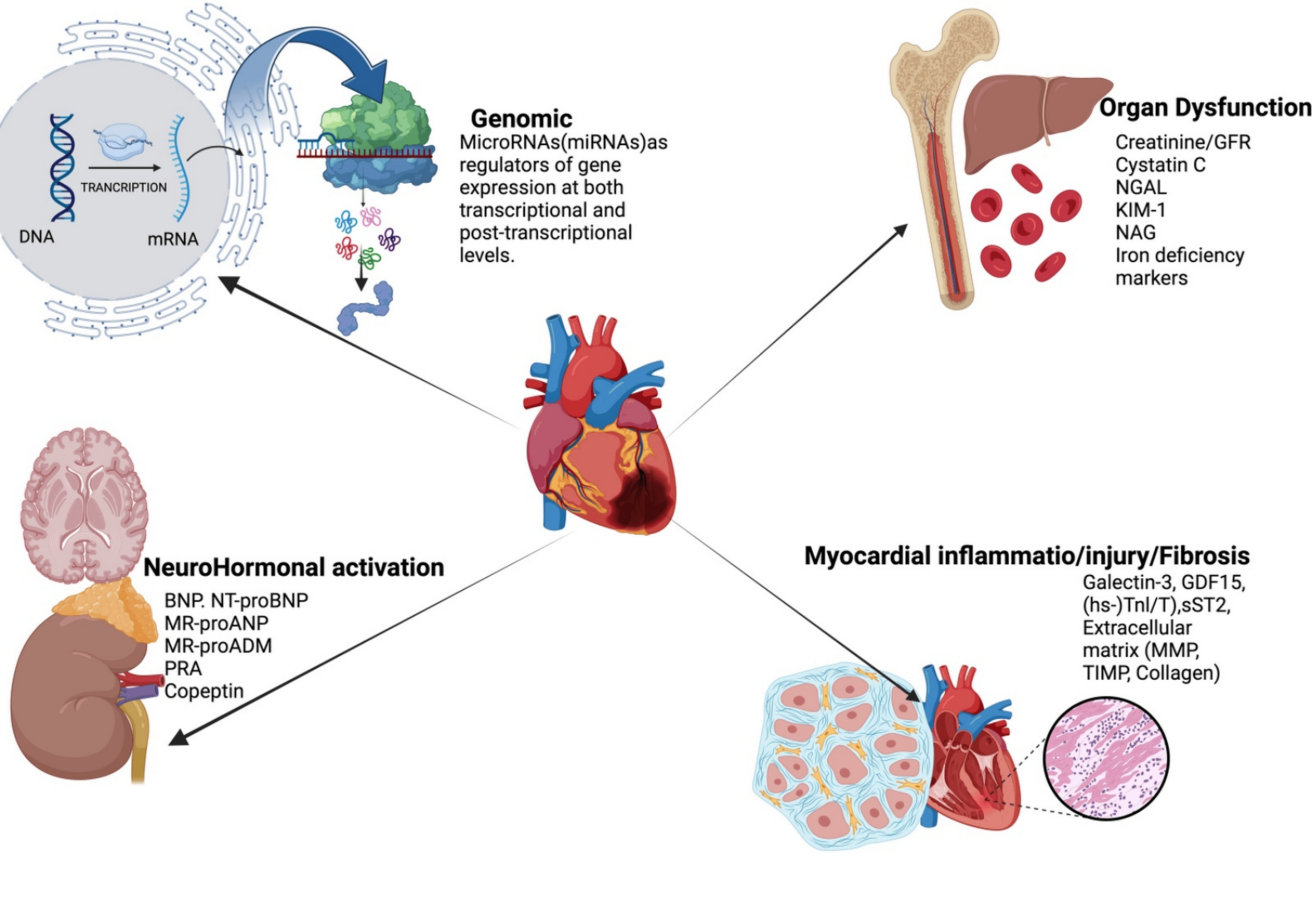
Pathophysiology of heart failure. Created using biorender.com. This figure is the original work of the authors. BNP: B-type natriuretic peptide; NT-proBNP: N-terminal pro-B-type natriuretic peptide; MR-proANP: mid-regional pro-atrial natriuretic peptide; MR-proADM: mid-regional pro-adrenomedullin; GDF-15: growth differentiation factor-15; MMP: metalloproteinase; PRA: plasma renin activity; GFR: glomerular filtration rate; NGAL: neutrophil gelatinase-associated lipocalin; KIM-1: kidney injury molecule-1; NAG: N-acetyl-β-d-glucosaminidase; TIMP: tissue inhibitors of metalloproteinases.

Activation of the RAAS, together with chronic low-degree inflammation, is mainly responsible for LV hypertrophy. The acute effect of female sex hormones may offer myocardial protection. However, the cumulative effects over the long term are detrimental, with post-menopausal women having the highest prevalence of hypertension compared with men in the same age group. Furthermore, there is an alteration in calcium homeostasis and changes in the autonomic nervous system and baroreceptor function, leading to impaired heart rate variability and increased vascular tone. The old human heart has structural abnormalities that affect diastolic, systolic, global longitudinal strain, and atrial function. Age-related loss of myocytes is attributed to several factors, including necrosis, apoptosis, autophagy, energetics, telomere/telomerase biology, and the role of the renin-angiotensin system [29]. Figure 1 depicts the pathophysiology of HF.

Diagnostic modalities

Clinical Assessment

The primary evaluation of patients with HF entails comprehensive history-taking and physical examination. Although patients with HF exhibit various signs and symptoms, no singular clinical manifestation has demonstrated diagnostic efficacy for HF. These manifestations exhibit variable sensitivities and specificities, thus engendering clinical suspicion of HF and warranting further investigation. Patients with systolic and diastolic HF types present symptoms related to impaired filling or reduced cardiac output, resulting in congestion and diminished cardiac output. Symptoms of congestion encompass dyspnea, characterized by increased shortness of breath during mild to moderate physical activity, depending on disease severity [30]. Dyspnea on exertion, while susceptible (100%) to HF, exhibits low specificity (17%), suggesting its presence in patients with diseases other than HF, such as various pulmonary disorders like interstitial lung diseases or obstructive lung diseases. Orthopnea and paroxysmal nocturnal dyspnea demonstrate moderate specificity (81%), rendering them valuable for diagnosing the disease, having a low positive predictive value (2%), and indicating limited diagnostic utility in excluding the disease [31]. Symptoms associated with low cardiac output, including fatigue and lethargy, result from muscle hypoperfusion-induced premature release of muscle lactate. Alternative causes contributing to fatigue include anemia, endothelial dysfunction, and skeletal muscle structural and functional abnormalities, diminishing its specificity for diagnosis [32]. Symptoms indicative of cerebral hypoperfusion and abnormal sleep patterns encompass somnolence and confusion. Peripheral edema, prominent on physical examination, exhibits high specificity (86%) but low sensitivity (20%), serving as a reliable indicator for diagnosing the disease. Gallop rhythm and pulmonary rales signify rapid ventricular filling and pulmonary edema. A displaced cardiac apex demonstrates high specificity (95%) and moderate sensitivity (66%), thus proving a more effective diagnostic modality. Clinical signs such as right hypochondriac pain (liver distension), abdominal swelling (ascites), loss of appetite, malabsorption (bowel edema), jugular venous distention, and weight gain suggest a clinical picture more indicative of right HF, either primary or secondary to pulmonary hypertension (cor pulmonale) [31]. The Framingham criteria, encompassing components of initial evaluation, serve as a validated diagnostic tool for both systolic and diastolic HF. Despite exhibiting higher sensitivity for systolic HF (97%) compared to diastolic (89%), it remains an effective tool for ruling out HF.

Laboratory Tests

Testing for various biomarkers in the patient's blood is instrumental in confirming the diagnosis and elucidating the etiology of HF. One such biomarker, elevated in response to increased myocardial stress and volume overload, is BNP and its NT-proBNP. BNP proves highly valuable in differentiating CHF from non-cardiac etiologies in patients presenting with dyspnea. Patients with BNP levels below 50 pg/mL are doubtful to have HF, while levels exceeding 150 pg/mL moderately support the diagnosis (specificity 83%). BNP levels surpassing 100 pg/mL warrant further evaluation for cardiac pathologies in conjunction with additional signs and symptoms. Elevated BNP levels correlate with a poor long-term prognosis and increased mortality risk in HF patients [33,34]. Additional laboratory assessments aim to identify etiology and potential treatment-related adverse effects. Evaluating serum electrolyte levels, including potassium, helps identify electrolyte imbalances that may precipitate arrhythmias. Calcium and magnesium levels assess the risk of arrhythmias and potential side effects of diuretic therapy. Complete blood count (CBC) evaluates for anemia. Liver function tests detect hepatic congestion resulting from volume overload secondary to right HF. Renal and thyroid function tests exclude renal or thyroid dysfunction contributing to HF, weight gain, or volume overload. Troponin and creatine kinase-myocardial band (CK-MB) levels indicate myocardial ischemia or injury. Hemoglobin A1C levels and lipid profile assessment aim to identify comorbidities such as type 2 diabetes mellitus or coronary artery disease associated with HF [35,36].

Novel biomarkers: inflammation and fibrosis

sST2

The suppression of tumorigenicity 2 (ST2) is a part of the IL-1 cytokine receptor family and IL-33 to signal immune cells and to cardiomyocyte injury of tissue. These receptors are expressed in two forms: soluble and membrane bonds. According to studies, its utility is mainly prognostic of chronic and acute HF [37]. For HF diagnosis, the studies show conflicting results, with some studies concluding that its level can be elevated both in subjects with HF, healthy individuals, and even in subjects with other inflammatory conditions such as chronic obstructive pulmonary disease (COPD). Compared to natriuretic peptides, a classic biomarker, it displays low specificity [37,38].

On the contrary, soluble suppression of tumorigenicity 2 (sST2) holds a much higher prognostic value due to its ability to support a hierarchy of risk groups, specifically for fatal endpoints. A meta-analysis conducted at a large scale measured sST2 concentrations not only before the admission of patients but also before discharge. It concluded that elevated levels at either point were fiercely related to mortality associated with cardiovascular causes [37,39]. Several studies have cemented that episodic sST2 measurement can have more substantial prognostic effects. A study on 150 acute decompensated HF patients concluded that fluctuation in ST2 can be a strong indicator of mortality in the short term [37,40]. Another study of a similar nature likewise showed that acute HF patients who had elevated sST2 levels during hospitalization and displayed the pattern consistently during hospitalization held the highest mortality rate in the long run [41]. In many studies conducted on chronic HF, sST2 maintained its importance as a vital indicator of prognosis, too [37,42]. High levels of sST2 were directly correlated with the risk of failure of HF therapy in device-assisted patients as well as in patients on pharmacological therapy [37,43].

Galectin 3

Galectin is vital in several cellular functions, including its growth, apoptosis, fibrosis, and inflammation. It has been recognized to belong to the family of lectins that bind beta-galactoside [37,44,45]. It exerts its effects in nuclear and extranuclear parts of the cells but is produced in the cytoplasm [37,45]. Galectin 3 is identified explicitly for collagen one production due to its interaction with cardiomyocytes [37,46,47]. The pathophysiology underlying this process is believed to be due to the activation of myofibroblasts by galectin 3 [37,48].

Many clinical and cohort studies on HF patients show its elevation [37,49-51]. Further, these studies have successfully concluded that the blood level in HF patients is directly correlated to mortality because of all existing causes of cardiovascular events [37,52-58].

Myocardial Fibrosis and Collagen Synthesis Markers (PICP, PIIINP, CITP)

The role of fibrosis in myocardial restructuring is well-established in HF. Hence, many attempts have been made to identify molecules that could act as biomarkers of myocardial fibrosis. Serum amino-terminal propeptide of procollagen type III (PIIINP), serum carboxy-terminal propeptide of procollagen type I (PICP), and serum collagen type I telopeptide (CITP) are three biomarkers that have stood the trials of being correlated with the myocardial fibrosis histology [59-61]. Serum PIIINP is formed due to type III procollagen converting to type III collagen extracellularly. Hence, it is understandable that its concentration in blood is directly proportional to the amount of myocardial fibrosis. This is called myocardial collagen III volume fraction (CIIIVF). It is possible to measure this parameter with particular imaging techniques [37,62]. Recently, serum concentration has been proven to correlate with the severity of HF [37,63]. Extracellularly, when type I procollagen alters to type I collagen, PICP is formed due to this conversion. As this process is increased in HF patients, so does its serum concentration [37,64]. Studies have shown its direct relation to ventricular arrhythmia, ejection fraction reduction, and mortality. Its level can be affected by treatments such as mineralocorticoid receptor antagonists and loop diuretics [37,65,66]. The ratio of CITP to serum matrix metalloproteinase (MMP)-1 indicated collagen escaping degradation by MMP. Therefore, this can be used as a risk identifier for hospitalization in HF patients [37,62].

ET-1 and MPO

Endothelin-1 (ET-1) peptide has been well studied for its vasoconstrictive effects, urine formation, and water homeostasis [37,67,68]. It has a well-proven role in the pathophysiology of pulmonary hypertension. ET-1 elevation seen in chronic HF patients is indispensable in remodeling cardiomyocytes, worsening hemodynamics, and activating the neurohormonal cascade. A study points to ET-1's role in disease severity, clinical signs of fluid overload, low natriuresis, and as an HF patient one-year mortality prognostic indicator [37,67,69,70].

Leukocyte secretes myeloperoxidase (MPO), which is pivotal in many inflammatory processes that result in oxidative damage. It has a significant role in atherosclerotic plaque and remodeling of ventricles [37,71]. Its serum level has been directly correlated with the development of chronic HF and HF severity. However, its role in acute HF is not well-proven [37,72-75]. MPO and ET-1 are unique in the study of cardiac pathology as these two are the only proven markers of inflammation in the development of cardiotoxicity post-therapeutic treatment of malignancy [37,76,77]. The level of ET-1 has been seen to be increased after doxorubicin treatment along with creatine phosphokinase and serum lactate dehydrogenase, aiding in the development of doxorubicin-induced cardiomyopathy [1,40]. A study by Ky et al. [76] established the role of MPO levels as an indicator of cardiotoxicity development in the long run (i.e., >10 years) after malignancy treatment [73,77].

Growth Differentiation Factor-15 (GDF-15)

There is not much evidence suggesting the role of GDF-15 in the human body in general and HF in particular. However, its level increases in several renal, pulmonary, and cardiac diseases [37,78]. Its elevated level, along with other biomarkers, can be utilized to predict LV remodeling, systolic dysfunction, and mortality due to all causes of cardiovascular death [37,79,80].

Serum Free Light Chains (sFLC)

In the last few years, increased curiosity in endothelial dysfunction and its pathogenesis in IHD progressing to HF has led to the increasing study of sFLC. Attempts have also been made to find its correlation with HF to discover a novel cardio biomarker of HF. It is present in many biological fluids [37,81]. However, its increased level is of great significance in the diagnosis of pathology of an inflammatory nature [37,82]. The possibility of its involvement in the development of risk in cardiovascular pathology has been suggested by many researchers, including Bellary et al. [82].

In contrast, Shantsila et al. found it to be predictive of mortality in acute cases of HF [83-85]. Several other studies have also concluded its role as a mortality predictor with different co-morbidities [37,86]. However, the pathophysiology and direct relation still need to be clarified. Nevertheless, sFLC holds the potential to emerge as one of the novel biomarkers of HF [37].

Neurohumoral biomarkers

Adrenomedullin (ADM)

ADM belongs to the family of peptide hormones. The primary function of ADM is similar to other natriuretic peptides acting on vessels to control tone and dilation and maintain the structural integrity of the endothelium [37,87]. Volume overload is one of the primary well-studied triggers for ADM [1,88]. One of the studies successfully concluded that plasma level was on the rise in HF patients compared to the control group. The main drawback of this biomarker is that it has no clear distinction based on the etiology of HF (i.e., non-ischemic HF or ischemic HF) [37,89].

Copeptin

The hypothalamus synthesizes arginine vasopressin (AVP). It is commonly known as an anti-diuretic hormone. Its primary function is regulating plasma osmolality and serum sodium concentration. Due to its effect on sodium and osmolality, it is secreted to counter hypovolemic state and low cardiac output. It is challenging to trace AVP due to its short half-life [37,90]. Copeptin, a C-terminal inactive fragment of AVP, is stable and can be easily detected. Repeated studies have shown its strong predictive role in rehospitalization and mortality compared to other natriuretic peptides such as BNP and NT-proBNP [37,90,91].

Long Noncoding RNA and HF

"Genetic Junk" is the total RNA, not translated into proteins. Lately, it has been discovered that junk DNA produces molecules that are essential in regulating several mechanisms. Depending on nucleotide length, these noncoding RNAs (ncRNAs) are divided into small ncRNAs (<200 nucleotides) and long noncoding RNA (lncRNA)(<200 nucleotides) [37].

lncRNAs are complex in their structures, promote gene transcription and post-transcription, and regulate all biological events. One of the characteristics of lncRNAs that can be exploited to our advantage is that they are tissue-specific and vary between species. It also helps that lncRNAs are stable molecules with a long half-life, thus exudating the potential to be a non-invasive diagnostic cardio biomarker [37,92-94].

LIPCAR (long intergenic noncoding RNA predicting cardiac remodeling) is one of the first recognized lncRNAs. It was derived from mitochondria, and a study showed its presence in patients with faulty LV remodeling following myocardial infarction. Studies have shown its relation to adverse prognosis in patients with HF [37,95,96].

WISPER (Wisp2 super-enhancer-associated RNA) lncRNA is supported by cardiac fibroblast. It is essential in the survival, migration, and proliferation of fibroblasts. It also controls fibrosis by regulating the genes involved in this process (COL1A1, COL3A1, FN1, and aSMA) within fibroblast. Due to these properties, it has emerged as a lucrative target in patients with aortic stenosis [37,97].

lncRNAs, cardiac hypertrophy-associated epigenetic regulator (CHAER) and cardiac hypertrophy-associated transcript (CHAST), have also been studied due to their potential epigenetic roles in manipulating hypertrophy genes [37,98,99].

An experiment on diabetic rats muting ANRIL (antisense lncRNA in the INK4 locus) showed reduced oxidative stress, decreased inflammatory molecules, and improved cardiac remodeling [37,100,101].

MALAT1 (metastasis-associated lung adenocarcinoma transcript 1), responsible for cardiac fibrosis following myocardial infarction, has been proven to stabilize LV function by preventing cardiomyocyte apoptosis when silenced [37,102].

SENCR (smooth muscle and endothelial cell-enriched migration/differentiation-associated lncRNA) and MIAT (myocardial infarction-associated transcript) have been studied to predict LV remodeling in rats suffering from diabetes with heart failure with preserved ejection fraction (HFpEF) [37,103].

HEAT2 (heart disease-associated transcript 2) aids fibroblast proliferation, adhesion, invasion, and transmigration. HEAT2 can also manipulate histone H3K27me3 [37,104,105].

MHRT (myosin heavy-chain-associated RNA transcript) and NRON (ncRNA repressor of NFAT) regulate calcium levels intracellularly, and both lncRNA activities are upregulated in HF. MHRT activation triggered by oxidative stress prevents H2O2-induced apoptosis of cardiomyocytes and the chromatin-remodeling cascade of molecular machinery. Thus exerting a protective effect [37,106,107]. ANRIL, EGOT, H19, HOTAIR, TUSC7, RMRP, RNY5, SOX2-OT, and SRA1 have also been established to have a role in end-stage HF and can be exploited to study as diagnostic cardiac biomarkers of HF [108].

Electrocardiogram (ECG)

Electrocardiography (ECG) serves as a pivotal diagnostic modality in the initial assessment of HF, facilitating the identification of various pathological scenarios. Firstly, left ventricular hypertrophy (LVH) is characterized by increased R waves in leads V5 and V6 and elevated S waves in lead V1. LVH may indicate underlying conditions such as hypertrophic cardiomyopathy or hypertension, often associated with diastolic HF [35]. Inversion of T waves signifies myocardial strain and indicates a poorer prognosis. Secondly, myocardial ischemia is typified by ST segment depression or T wave inversion on the ECG. Enlarged and widened Q waves may also suggest ischemia or previous infarction. Thirdly, right ventricular hypertrophy (RVH) is discerned by a dominant R wave in leads V1 and V2, alongside abnormal axis deviation, potentially suggesting right HF due to conditions such as cor pulmonale, pulmonary embolism, or pulmonary hypertension. Fourthly, arrhythmias manifest in various forms: the absence of P waves with regular QRS rhythms suggests paroxysmal supraventricular tachycardia, while the absence of P waves with irregular QRS rhythms indicates atrial fibrillation [35]. A sawtooth appearance of P waves characterizes atrial flutter. Lastly, ECG aids in detecting medication side effects and electrolyte imbalances. Certain medications like adenosine and digoxin may induce heart block, which is evident by a prolonged PR interval. Tricyclic antidepressants can prolong the QT interval. Rapidly progressing hyperkalemia is identifiable by tall, tented T waves on the ECG, progressing eventually to sine waves, representing a medical emergency [35].

Echocardiography

Echocardiography is a fundamental diagnostic tool for confirming the diagnosis of suspected systolic HF. It evaluates cardiac structure by measuring LV dimensions, wall thickness, volumes, and function through parameters such as ejection fraction and mitral flow velocities. Systolic HF is readily discernible on echocardiography, evidenced by an enlarged left ventricle with reduced ejection fraction (<40%). Ejection fraction values between 40% and 49% denote HF with moderately reduced ejection fraction, following a similar treatment algorithm for HF with reduced ejection fraction. Ejection fraction exceeding 50% prompts assessment of other aspects of LV structure and function to confirm HF with preserved ejection fraction. The primary diagnostic indicator for diastolic HF is the E/A ratio. E represents peak velocity through the mitral valve during early diastole, and A represents peak velocity during late diastole (atrial contraction phase). In the early stages of diastolic HF, the E/A ratio decreases due to a loss of pressure gradient across the mitral valve. As the disease progresses, increased pressure is transmitted to the left atrium, restoring the pressure gradient and pseudonormalizing the ratio. Advanced stages present with a restrictive HF pattern [109]. Several additional parameters support the diagnosis of diastolic HF: increased wall thickness serves as an indicator of LVH, which may be secondary to conditions such as hypertension, obesity, or diabetes, or it may signify hypertrophic cardiomyopathy. Left atrial enlargement can result from diastolic dysfunction but may also occur due to atrial arrhythmias or mitral valve disorders. Elevated estimated pulmonary pressure may indicate right ventricular failure secondary to pulmonary causes (cor pulmonale) or, more commonly, result from decreased forward flow of blood due to LV failure [110].

One limitation of two-dimensional echocardiography (2D echo) is its subjective assessment of LV size and function, leading to significant interobserver variability and potential inaccuracies in diagnosis [110]. Several three-dimensional echocardiography (3D echo) techniques have become available to address this limitation. 3D echo does not rely on geometric assumptions for volume/mass calculations and is not susceptible to errors in plane positioning. Studies have demonstrated that 3D echo is more accurate, with significantly better agreement and lower interobserver variability than 2D echo.

Myocardial viability assessment is crucial in determining response to therapy and prognosis. Stress echocardiography evaluates myocardial viability by assessing contractile reserve. Indices such as ejection fraction, wall motion index, and force-frequency relationship measure contractile reserve. An increase in ejection fraction by more than 5% or a change from baseline by more than 20% during stress echocardiography indicates good myocardial reserve and, hence, a better prognosis in patients with diastolic HF. However, ejection fraction measurement has limitations and should not be used as the sole indicator for viability assessment. Heart transplantation is the inevitable option for HF patients without myocardial viability during stress echocardiography [110].

Cardiac Magnetic Resonance Imaging

Cardiac MRI is essential for imaging heart structure, function, and myocardial tissue composition. It is recommended to evaluate HF and assess ventricular function/mass due to its high spatial resolution and ability to quantify heart anatomy accurately [111].

Although echocardiography and computed tomography are usual diagnostic partners, cardiac MRI offers a recognized added differential diagnostic benefit in the comprehensive HF workup. Cardiac MRI (CMR) offers a comprehensive, noninvasive assessment of cardiac structure, function, tissue characterization, and perfusion in patients referred for HF assessment [112,113].

Cardiac MRI can visualize various aspects of the cardiac structure, including the myocardium, both right and left ventricles, atria, and myocardial valves. Cardiac MRI can provide information about the structural and functional anatomy of the heart and great vessels in patients with congenital heart disease. It is particularly well-suited for imaging myocardial tissue and its ability to distinguish tissue such as blood, fat, iron, and myocardium. Techniques used to visualize the myocardium include T1-weighted, T2-weighted, T2*-weighted perfusion, and late gadolinium enhancement (LGE) imaging. These techniques allow the diagnosis of various myocardial pathologies, including myocardial iron loading, edema, ischemia, and fibrosis. Moreover, myocardial perfusion and strain imaging are used to detect myocardial ischemia and to quantify regional or global myocardial function, respectively. Cardiac chamber components can be visualized throughout the cardiac cycle, including the end-diastolic and end-systolic volumes of the left and right ventricles (end-diastolic left ventricular volume (EDLV) or LV and end-diastolic stroke volume (EDSV) or stroke volume, respectively) [114].

The ability of cardiac MRI to noninvasively and accurately image these varied functions also provides an invaluable research and diagnostic tool for pediatric and adult patients with congenital heart diseases and acquired vascular and myocardial diseases [115].

The primary importance of the assessment of ventricular function of patients with suspected HF is to quantify LV systolic function and to determine if the patient has signs of diastolic or systolic HF, or both, incomplete rupture of the myocardium due to specific causes such as myocardial infarction due to Takotsubo syndrome or myocardial infarction with cardiac rupture, multichannel-specific T1 mapping can be used to distinguish the diagnosis. The area of tissue characterized by a significant MRI signal increase and T1 relaxation reflects the area of myocardial fibrosis. T1 mapping can also identify the affected myocardium in cases of hypertrophic cardiomyopathy by using extracellular volume characterization and mapping in cases of myocarditis [116,117]. The large field of view of cardiac MRI can be achieved without ionizing radiation [117].

The advent of three to five-element timings has significantly accelerated 3D cine sequences. Additionally, the introduction of 4D flow CMR has opened up exciting new opportunities to model the patterns of blood flow, which may not only aid with the stratification of cardiovascular disease but may provide important insights for personalized surgical planning. Due to its high-contrast capabilities, CMR has the potential to be used as a research tool for image-based diagnostics and therapeutics; for example, dyeing or scar-specific agents used to map myocardial fibrosis load or energies can be used to trigger gene delivery for local overexpression or inhibition of target receptors [118].

The clinical indications for cardiac MRI in HF are similar to those for echocardiography. For patients with less than optimal echocardiogram images or poor acoustic windows, cardiac MRI may be used for cardiac chamber volume measurements, functional assessment, and to visualize myocardial fibrosis. The addition of LGE helps diagnose diseases affecting the myocardium, such as myocarditis, sarcoidosis, amyloid accumulation, or myocardial fibrosis from ischemia. Measuring cardiac volumes allows for assessing ventricular dysfunction and can help guide HF management. The changes in diastolic volumes, stroke volumes, and end-systolic volumes of the left ventricle indicate how the heart changes ventricular volumes in response to afterload stress with changes in preload [119]. Each image is visually assessed, and then calculations are carried out automatically or interactively using dedicated software. Essential imaging can assess myocardial edema, transplant rejection, myocardial infarction in the acute phase, and pericardial effusion and cardiac MRI angiography [120].

Cardiac mass is increased in hypertrophic hearts. MRI offers a fast and reproducible way to obtain cardiac mass on routine MRI examination and is less operator-dependent than echocardiography [121]. The general role of MRI in the evaluation of cardiac mass and wall thickness includes four ways: fast and accurate determination of LV mass and wall thickness; accurate detection of conditions characterized by LVH such as hypertensive heart disease, hypertrophic cardiomyopathy, aortic stenosis, and athleticism; detection of segmental or transmural wall hypertrophy in arrhythmogenic right ventricular dysplasia; and calculation of the proportion of LV mass due to LVH by using common findings of LV masses specific for age, sex, and body surface area, as calculated by the MRI. In conclusion, after a standard cardiac MRI protocol, images are obtained in every patient. These pertain to evaluating the ventricle or both ventricles in short-axis images and the myocardial mass and wall thickness of the right ventricular (RV) free wall in RV-focused short-axis images. Post-image processing for mass and wall thickness in hypertensive heart disease, hypertrophic cardiomyopathy, arrhythmogenic right ventricular cardiomyopathy (ARVC), and the proportion of tremendous LVH can be easily performed by a board-certified radiologist by comparing it with the latest average measurements from the literature [122].

LGE reflects only the scar patterns. It will not map the distribution or quantify the amount of fibrotic tissue in those without overt myocardial damage. The newer LGE provides a precise quantitative estimation of focal fibrosis, mainly due to myocardial scarring in ischemic and non-ischemic cardiomyopathies. Moreover, the evolution of myocardial extracellular volume and effective transverse relaxation time mapping might be valuable tools to assess different types of myocardial diffuse fibrosis, what roles they would play in the future, and T1 and T2 mapping [123].

Acute inflammation and non-fibrotic scar tissue also lead to the detection of myocardial enhancement with T1 maps. However, high variation indices mean T1 maps are unsuitable for frequency-modulated bus sequences and surface coil intensity correction. A possible alternative is fast pixel-wise myocardial maps and quantification during healthy heart motion. A short transmural fibrosis assessment underscores the clinical relevance of T1 mapping to detect fibrosis detection. T1 mapping uses manganese instead of gadolinium for myocardial microfibrosis detection but is less successful. T1 mapping techniques used in cardiac imaging, including those sensitive to radiofrequency pulses and changes during ischemia, and methods like 3D just encompassing reduction for identifying myocardial scar tissue, are effective in detecting certain heart tissue abnormalities. However, they are not sensitive enough to detect microvascular obstruction, which involves blockages in the small blood vessels of the heart [124].

Myocardial fibrosis can be assessed with various MRI techniques, such as late enhancement imaging using T1-weighted imaging after gadolinium injection. This technique requires relatively large areas of fibrosis with extension into healthy myocardium to be detectable. While myocardial fibrosis in dilated cardiomyopathy is usually found in regions with previous myocardial infarction, ischemic cardiomyopathy typically features transmurality, subendocardial involvement, and involvement at the periphery of the LV. Furthermore, biochemical static MRI methods can detect low myocardial fibrosis. Additional information regarding diastolic function can be inferred when the extracellular volume is analyzed [124,125].

A first-pass perfusion MRI assessment of myocardial blood flow for detecting viable myocardium has been examined compared to positron emission tomography (PET) and single-photon emission computed tomography (SPECT). In the first-pass perfusion series, stress hypo-perfusion of contrast material is observed in the areas with myocardial ischemia. In a comparison between the MRI and PET methods (rest myocardial blood flow, ^13NH3 PET) using an animal experiment (15 VX2 tumor-bearing rabbits), the rest blood flow evaluated by first-pass perfusion MRI showed a good correlation with PET (r = 0.84). The capability of judging myocardial viability by 3.0 T is enhanced. The result of detecting a previous myocardial infarction or myocardial ischemia is reported to be better, 3.0 T/0.6 mm (90/142) compared with 1.5 T/0.75 mm (76/142). At 3.0 T, it is necessary to suppress infragingival fat or to correct it with an algorithm in case of including fat in the myocardial contour; however, 3.0 T MRI provides rich information within a short examination time. Virtual AHA (American Heart Association) and Look-Locker techniques are used in cardiac MRI to enhance the detection and characterization of myocardial tissue abnormalities, including previous myocardial infarction or ischemia. The results in relation to mitral valve opening are reported to be superior. At 3.0 T, the TI value could be ideally set because of rich data. It is essential to judge contrast-enhancement characteristics because of the effects combined by contrast material dose and glomerular filtration rate (GFR) [126].

Focusing more on cardiac MRI alone can significantly improve left ventricular ejection fraction (LVEF) if a significant portion of the myocardium remains viable. Cardiac MRI is increasingly and successfully used for this purpose. Wall motion and thickening are judged in both the cine and LGE images, and there are several specific MRI methods to judge viability. These methods include evaluating myocardial blood flow, first-pass of contrast material, myocardial contrast echo/cardiac resynchronization therapy (CRT), PET, SPECT, and ultrastructural information such as inflammation and fibrous tissue. A perfusion stressing examination is necessary to decrease the cardiac motion or width of the slice for the perfusion series [127].

In some clinical scenarios, high-quality ECG-gated dobutamine stress cardiac MRI can ascertain inotropic reserve and identify myocardial viability more accurately than delayed enhancement MRI. For this reason, pharmacological stress cardiac MRI is achieved with dobutamine administration. However, stress cardiac MRI is also performed with the damaging contrast agent regadenoson (Lexiscan) in patients with contraindications to dobutamine use, including the presence of arrhythmias, severe valvular heart disease, and significant stenotic coronary artery disease. Regadenoson is an A2A adenosine receptor agonist. Healthy, normal myocardium does not contain adenosine A2A receptors, so myocardial blood flow is increased through coronary artery vasodilatation in areas with myocardial viability. However, there is no heterogeneity in vasodilatory response, resulting in no stress hypoperfusion. Finally, it should be remembered that myocardial hibernation demonstrates blunted muscle inotropic response with low-dose dobutamine. The ECG should be carefully monitored while adenosine receptor agonists and other vasodilators are given [128,129].

Cardiac MRI can assess myocardial viability through delayed enhancement MRI, which can visualize myocardial scar because of its propensity to have prominent extracellular space. This can help determine the degree of myocardial viability for predicting improved myocardial contractility following revascularization. There are several causes of nonviable myocardium seen on delayed enhancement MRI. It is important to remember that focal myocardial scar due to healed myocarditis also marks nonviable myocardium. The volume of nonviable myocardium can be estimated by threshold techniques to calculate infarct size as a percentage of total LV mass [130].

Nuclear Imaging Techniques

Nuclear medicine techniques provide crucial noninvasive tools in diagnosing and managing HF, offering functional and molecular insights into cardiac physiology. Traditional methods like SPECT and PET have been pivotal. However, recent advances have significantly expanded the diagnostic capabilities of nuclear imaging in HF [121].

Recent developments in PET imaging involve novel radiotracers that target specific molecular pathways involved in HF. Fluorine-18 labeled tracers, such as 18F-fluorodeoxyglucose (FDG), are commonly used to assess myocardial viability. Enhanced FDG uptake in myocardial cells signifies viable but hibernating myocardium, guiding revascularization decisions. Moreover, emerging tracers like 18F-flubrobenguane are being investigated for their potential to map sympathetic innervation, offering a new dimension in evaluating the risk of arrhythmias and sudden cardiac death in HF patients [121].

PET/MRI hybrid imaging: The integration of PET with MRI offers simultaneous functional and morphological data, enhancing the diagnostic accuracy in HF. In a single session, PET/MRI hybrid imaging allows for detailed evaluation of myocardial scarring, perfusion, and metabolism. This technique is precious in differentiating ischemic from non-ischemic cardiomyopathy by combining the metabolic insights from PET with the high-resolution tissue characterization capabilities of MRI [131,132].

Advanced SPECT techniques: Technological advancements in SPECT imaging, such as cadmium-zinc-telluride (CZT) cameras, provide higher resolution and faster acquisition times, facilitating more precise assessments of myocardial perfusion and function. These cameras allow for dynamic imaging and are capable of capturing myocardial blood flow and reserve quantitatively, similar to PET. Such features are crucial for assessing the severity and extent of IHD underlying HF [132].

Cardiac neuronal imaging with iodine-123 metaiodobenzylguanidine (123I-mIBG) reflects sympathetic nervous system integrity and is prognostically significant in HF. A reduced heart-to-mediastinum (H/M) ratio of 123I-mIBG uptake is associated with an increased risk of cardiac events. This imaging modality is increasingly used to refine risk stratification and guide therapy in HF patients, especially using devices like implantable cardioverter-defibrillators (ICDs) [123].

Quantitative blood flow measurement: Quantitative techniques that measure myocardial blood flow and reserve are gaining traction, mainly using PET. These methods provide critical insights into microvascular dysfunction, a common feature in HF, even when major coronary arteries are not significantly obstructed. Quantifying myocardial blood flow helps in understanding the subtleties of HF pathology, particularly in HF with preserved ejection fraction, where coronary microvascular dysfunction plays a significant role [132].

Cardiac Catheterization and Hemodynamic Assessment

Cardiac function evaluation involves assessing both external heart mechanics and internal heart function. Invasive procedures are often used to evaluate the heart's function. However, measuring systole decrease for accurate assessment requires precise and reliable clinical procedures, which are currently limited. Assessing the intensity and timing of myocardial motion is essential but needs to be more utilized due to the skill required for interpretation [133].

Invasive heart function assessment is crucial for understanding cardiac diseases. It precedes invasive treatment and helps minimize anesthesia and ischemic times. It also aids in evaluating ventricle repair techniques, stem cell transplantation, and ventricular regenerative capability. Physical assessment measures pressure, workload, volume, and heart rate to develop new techniques for diagnosing and treating cardiac patients. Evaluating and comparing ventricle repair techniques in a dynamic environment is essential. Physical assessment includes measuring pressure, volume, heart rate, and workload. These tools have advanced cardiac patient diagnosis and treatment [134].

The practice of invasive ventricular pressure-volume measurements has provided clinical investigators with insights into HF and its response to therapy. Direct measurement of LV end-diastolic pressure (EDP) and the end-diastolic pressure-volume relationship (EDPVR) offers confusing insights. A miniature pressure-volume catheter allows for routine ventricular space-time mapping. Based on physics principles, the measurement of LV EDP and EDPVR provided valuable insights. Laws of physics describe the contributors to myocardial relaxation and ventricular filling [135].

Cardiac catheterization is the gold standard for directly assessing the LV EDP, which is the critical parameter to diagnose HF in patients presenting at clinical examination with symptoms and signs suggestive of this syndrome. Although this technique has been improved, it remains invasive. It is not suitable to screen patients with unclear but suggestive clinical presentations or evaluate therapeutic interventions in patients with or at risk for the syndrome [136].

Invasive hemodynamic assessment is performed using a catheter system that is introduced into the heart and the circulation. Through this catheter system, it is possible to measure different parameters of interest. These ultimately give information about the function of the left heart (LH) or right heart (RH), the pump function of the heart, or the filling pressures and hemodynamic parameters at various points throughout the circulation. Nevertheless, invasive hemodynamics has three necessary restrictions: pressure is relative, not absolute, measured at only one point, and characterized through a punctual instantaneous value with no data tracked over time [137].

Hemodynamic phenomena are quantitated and assessed in patients with HF to gain relevant pathophysiologic and diagnostic information. Accurate measurement of cardiac output, pulmonary and systemic vascular resistances, and intracardiac pressures contribute significantly to understanding the basis of HF and the ability to predict the outcomes that follow the institution of pharmacologic or other management [138].

Cardiac output is central to the hemodynamic derangement in HF. Measurements of right- or left-sided filling pressures (proper atrial pressure, pulmonary capillary wedge pressure, or LV EDP) are necessary to determine if the degree of HF symptoms can be attributed to elevated filling pressures; adjustment of physician decisions based on these pressures can decrease symptoms and enhance quality of life. Additional hemodynamic understanding is required if vasodilator treatment of less than optimal efficacy is planned; cardiac index, systemic vascular resistance, or mixed venous oxygen saturation are frequently monitored. Data collected from the Swan-Ganz catheterization technique can provide information on central blood and oxygen parameters, generally using a thermodilution technique that also determines cardiac output. Although these measurements are core to patient assessment and predict outcomes, they represent only part of HF's overall cardiac, pulmonary, and systemic artery mechanisms. More sophisticated evaluation of the unique physiologic events involved in HF is warranted, as newer pharmacologic or mechanical therapy often targets more discrete physiologic derangements. Thus, the Swan-Ganz catheter represents only a portion of what is needed to understand hemodynamic derangements in these patients [139,140].

By morphological assessment, CT allows a large field of view and a 3D assessment of the complex heart and vascular structures. From a structural measurement perspective, cardiac CT has yet to be fully utilized. Since cardiac CT images are acquired through a scan-obligated temporal resolution, which may blur the moving heart, the temporal resolution-centric imaging strategy has dominated the last few decades of advances in CT technology. Since 256-640 detector row CT has demonstrated improvement in axial-array coverage, volumetric heart rates approaching faster than the intrinsic heart rate has started to blur the problem of temporal resolution in select cases. With half the radiation dose or scanning time used to achieve faster volumetric heart rates, the main advantage of the wide X-ray beam or virtual dual-source configuration of a 320-row CT is the capability to perform high-resolution dynamic volumetric imaging for the entire heart and thoracic aorta, which may lead to innovative investigations and breakthroughs of cardiac or cardiovascular functions [141].

Three-dimensional and bifurcation capabilities are most recently available and offer advantages over standard systems for the physician and the patient. As cardiac computed tomography becomes involved in the diagnostic workup for HF, antiarrhythmic drugs and up-titration of guideline-driven HF treatment should be considered. Sedentary stress testing is evolving and has the potential to offer a direct and noninvasive examination of perfusion and valve function in HF, which has not previously been available. Categories of patients who may be more suited to sedentary stress testing are elderly patients, who may not be able to consent to specific interventions, pacing-dependent right ventricular pacing, claustrophobic patients, and those with prior allergic reactions to iodinated contrast [142].

Stress tests have played a central role in the diagnosis of HF as well as in prognostication. It involves measuring the fraction of CO2 and oxygen in inspired air. In contemporary practice, the utility of pharmacological stress testing is, however, limited by adverse reactions and contraindications associated with vasodilators and inotropes such as dobutamine. Additionally, pharmacological stress testing carries an increased risk of provoking tachyarrhythmias and hypotension in the often fragile HF patient cohort, which may require stabilization prior to the undertaking of the test. In many centers, the availability of cardiology support during this procedure limits the ability to perform testing promptly [143]. Sedentary scanning systems have shown promise in the literature and have gained wider acceptance, as they can offer reduced radiation doses and improved image quality [144,145].

Over the last several years, significant developments have been made in imaging modalities in the evaluative phase for acute and chronic HF, mainly in MRI and PET. This review focuses on recent advancements in CT scans and emerging technologies, mainly on signal transduction using approved imaging modalities or molecular and molecular imaging. In the future, cell science will use molecular imaging, the visual direct visualization of inflammation by the activation of framers, and the evaluation of metabolic imaging, which can become a new frontier that these techniques have analyzed. However, they have not yet been touched [146].

We will review the potential of different diagnostic imaging modalities, namely, multislice computed tomography (MSCT), advanced echocardiography, and cardio-oncology tools, in HF. Quantitative morphological and functional imaging parameters obtained from MSCT can be used to detect and stage heart failure. Despite the eminent need for burgeoning diagnostic imaging developments to accurately stage HF, whether in pre-existing conditions (such as valve disease, IHD, diabetes, obesity, hypertension) or in "incidental" HF-detectable conditions (such as cancer) in multimorbid, pluritherapic, evermore aging, increasingly survivor patients, and to mark the borders between comorbidities and HF, as well as the demarcations between different HF staging grades, integrated multitasking diagnostic imaging is currently grossly under-boarded in the HF field [147].

Challenges in diagnosis

Differentiating Heart Failure From Other Causes of Similar Symptoms

The accurate diagnosis of HF is a major clinical challenge, as the condition often shares symptoms with other cardiorespiratory diseases. A significant problem is distinguishing HF from other causes of exercise intolerance and dyspnea, such as COPD or interstitial lung disease.

Patients could use their symptoms as indicators of their ailments. Patients suffering from HF frequently experience a variety of symptoms, such as fatigue, peripheral edema, and dyspnea upon exertion. These symptoms might also arise in individuals who are clinically stable due to the chronic and progressive nature of HF. Nonetheless, one crucial early sign of decompensation is a shift in the frequency or intensity of these symptoms. Patients with HF frequently struggle to identify their symptoms, identify the underlying cause of their symptoms, and distinguish HF symptoms from those of concomitant diseases or aging. Prior hospitalization for HF does not improve patients' ability to respond promptly to their symptoms [148,149].

A study details how the perception of symptoms is multidimensional, meaning they are more than just a physical sensation, in line with the notion of unpleasant symptoms. The possible combinations of several symptoms are also taken into account by the theory [150-152]. The three-tier theoretical model explains the impact of antecedent physiological, psychological, and environmental factors on symptom features and patient response. Weight gain, the onset or worsening of peripheral edema, increasing dyspnea with exertion, an increase in abdomen circumference, and exhaustion are early signs of imminent decompensated HF. If medical attention is not received, these symptoms frequently worsen and eventually lead to more physically demanding symptoms like severe dyspnea, which makes it challenging to speak in complete sentences; paroxysmal nocturnal dyspnea, which causes difficulty breathing while sleeping; or orthopnea, which makes it difficult to breathe when a person is lying flat. Several typical causes of decompensated HF include infection, indifference to salt, and noncompliance with medicine [153]. It might be difficult for patients to be sensitive to and recognize a slight but subtle increase in the severity of HF symptoms (such as reduced activity tolerance) from chronic baseline symptoms to decompensated HF.

Limitations and Pitfalls of Current Diagnostic Modalities

To understand the significant limitations of current diagnostic modalities of HF, it is essential to understand the complexity of HF. The pathophysiology involves the involvement of heart chambers and multisystem involvement, which plays a vital role [154]. With the progression of time, HF becomes a disease involving several systems of the body and, ultimately, death. Current diagnostic modalities focus heavily on LVEF for risk evaluation, classifications, and therapeutic management [154].

The classification of HF based on LVEF has a plethora of limitations. To begin with, it disregards the primary mechanism and specific etiology of HF. Secondly, LVEF is mostly load-dependent and can present various pictures by the same patient in imaging, depending on their hemodynamic conditions or existing comorbidities [154]. For example, mitral valve prolapse can overestimate LVEF. Considering the same principle, a hypertrophic heart disease might show normal or higher LVEF. It discards the consideration of dysfunctional myocardium [154].

When the echocardiographic point of view is considered, LVEF is just one of the many factors involved; hence, it becomes essential to consider other parameters too. This is particularly important with the growing prevalence of HFpEF in the last few years [154].

Limitations of Pharmacotherapy

Several continuous studies and trials on HF patients, including end-point hospitalization of a progressive nature or worsening in nature, have been conducted using the "gold standard" of survival [155]. These studies and trials have shown that only beta-blockers and ACE inhibitors are effective in consistent survival advantage over time amongst the plethora of drugs used to manage HF in both acute and chronic conditions. These two drugs remain the foundation of current guidelines for managing HF. It is also noteworthy that although angiotensin II receptor blocker (ARB) use does not show any survival advantage over beta-blockers and ACE inhibitors, it still serves as a helpful alternative in several patients [155].

Integration of Diagnostic Modalities

Roughly half of the individuals diagnosed with HF exhibit a normal LVEF, leading to a classification known as HFpEF [156]. This has impacted the diagnosis and therapy of HFpEF. Thus, by harmonizing diverse imaging modalities, clinicians gain a holistic understanding of each patient's unique condition, shaping treatment strategies and risk assessment beyond mere diagnosis and facilitating well-informed decisions [157]. By relying on various imaging modalities, the depth of diagnostic information available to the physician increases, thus allowing for a greater understanding of the morphofunctional abnormalities of the disease in question and a tailored plan of action for each patient. The benefits are limited to diagnosis, risk stratification, management, monitoring after cardiac pharmacology or electrical therapy, and rehabilitation [158]. Clinical decisions regarding the management of HF often hinge on assessing LV function, primarily through transthoracic echocardiography (TTE) and transesophageal echocardiography (TEE). These diagnostic tools are typically accessible in primary care settings. However, this reliance on TTE and TEE can prolong the diagnosis and decision-making process for acute heart failure (AHF), leading to extended hospital stays and frequent costly visits to outpatient clinics. Consequently, the window for early AHF detection is frequently missed. The availability of alternative imaging modalities like cardiac computed tomography (CCT), nuclear imaging, and CMR in specialized centers becomes crucial for evaluating complex cases, potentially impacting the survival rates of AHF patients [159].

Future directions

Artificial Intelligence in Heart Failure Diagnosis

Despite the surplus of diagnostic modalities available today, diagnosing HF clinically can be challenging. Artificial intelligence (AI) uses computer programming to mimic human cognition and can evaluate large datasets through pre-programmed instructions to guide clinical decision-making with or without human supervision [160]. Recent advancements in AI have propelled its potential as a diagnostic and prognostic tool in HF. A subset of AI, machine learning (ML), uses models to interpret patterns from data, process them, and develop new algorithms to perform designated tasks. These ML-based systems are designed to learn from the data provided, enabling them to make decisions based on the datasets they are trained on [161]. Currently, ML has the potential to be applied clinically in several ways, most easily through clinical decision support systems (CDSS) and pattern recognition.

Pattern recognition is a significant attribute of AI technology. Numerous models have been used in various cardiac imaging modalities such as an echocardiogram, CT, and MRI for accurate and efficient evaluation of heart disease [162,163]. These models can learn and recognize subtle signs of pathology comparable to board-certified cardiologists. One model in particular has used echocardiographic images to predict LVEF in patients with HF [164]. Moreover, the accuracy of AI-assessed LVEF may be equal to that of expert readers [165]. Models like this may be reliable in assessing HF in clinical settings. Combining echocardiography with AI software to interpret HF, among other pathologies, may speed diagnosis and improve patient outcomes, especially in resource-deprived areas.

ECG is commonly used to diagnose cardiac ischemia and arrhythmias. Several studies have shown that ECG findings may help diagnose HF [166,167]. Augmenting ECG with AI interpretation can be a robust screening tool and identify early LV dysfunction in asymptomatic patients [166,167]. In particular, a highly accurate model was developed by analyzing 55,163 ECGs from 22,765 patients with HF. The model showed an advanced interpretation of ECGs to identify heart failure with reduced ejection fraction (HFrEF) and HFpEF [168].

CDSS are computerized tools that assist physicians in making a prompt and accurate diagnosis [169]. ML models can be incorporated into a CDSS to assist clinicians in diagnosing HF. These systems can aid in diagnosis and risk stratification and provide treatment recommendations. Recently, a study evaluated the utility and accuracy of an AI CDSS in assisting physicians in diagnosing HF [170]. The ML model integrated various patient parameters such as LVEF, clinical features, physical exam findings, and ECG features. The AI CDSS showed high concordance rates (98%) compared to HF specialists. Non-HF specialists had lower diagnostic accuracy (76%) than HF specialists. Furthermore, AI CDSS was able to stratify patients into HFrEF, heart failure with mildly reduced ejection fraction (HFmEF), and HFpEF subclassifications [170]. Hence, these ML-based networks can support physicians in making timely diagnoses, developing risk assessments, and planning appropriate medical therapy, especially in clinical settings where HF specialists may not be available.

AI's role in HF diagnosis is promising but needs to be clarified, as AI-based technologies still need to be improved to replace the expertise of experienced physicians. Furthermore, a notable limitation of AI models is the "garbage in, garbage out" phenomenon due to entraining AI models with inaccurate and biased datasets [170]. Introducing bias may stall the adoption of AI in several levels of clinical practice. However, more advanced models, such as deep learning algorithms, avoid bias altogether by not requiring entrainment or supervision. Nevertheless, trained experts must validate and evaluate assistive AI models like an AI CDSS before integration into the clinical workflow.

## Conclusions

HF can present acutely or progress over time. At present, due to the concern of a large population being already affected or falling in the strata of high-risk groups, several diagnostic modalities to predict and diagnose HF are a lucrative research target. Moreover, rapid evolution and discovery in this field, such as novel cardiac biomarkers, echo imaging, cardiac MRI, and nuclear imaging techniques, have aided curiosity further.

To hammer the nail on the head, the exponential development in the application and its consequence and influence in HF demands an increase in the expertise and knowledge in interpreting and integrating the information provided by diagnostic imaging by the multidisciplinary team involved. Intimate co-ordination seeks to understand and develop diagnostics in other potentially related quantitative, translational, and computational multi-omics approaches, and it is expected from the clinical specialists to lead to a growth in knowledge, novel insights, and management criteria, which will participate in the development of diagnostic driven advancement toward the clinical practice of the future.

## References

[1] Wagner S, Cohn K (1977). Heart failure: a proposed definition and classification. Arch Intern Med.

[2] Yancy CW, Jessup M, Bozkurt B (2013). 2013 ACCF/AHA guideline for the management of heart failure: a report of the American College of Cardiology Foundation/American Heart Association Task Force on practice guidelines. Circulation.

[3] Ponikowski P, Voors AA, Anker SD (2016). 2016 ESC guidelines for the diagnosis and treatment of acute and chronic heart failure: the task force for the diagnosis and treatment of acute and chronic heart failure of the European Society of Cardiology (ESC). Developed with the special contribution of the Heart Failure Association (HFA) of the ESC. Eur J Heart Fail.

[4] Tsutsui H, Isobe M, Ito H (2019). JCS 2017/JHFS 2017 guideline on diagnosis and treatment of acute and chronic heart failure - digest version. Circ J.

[5] Seferović PM, Vardas P, Jankowska EA (2021). The Heart Failure Association Atlas: heart failure epidemiology and management statistics 2019. Eur J Heart Fail.

[6] Heidenreich PA, Albert NM, Allen LA (2013). Forecasting the impact of heart failure in the United States: a policy statement from the American Heart Association. Circ Heart Fail.

[7] Timmis A, Townsend N, Gale C (2024). European Society of Cardiology: cardiovascular disease statistics 2017. Eur Heart J.

[8] Savarese G, Becher PM, Lund LH, Seferovic P, Rosano GM, Coats AJ (2023). Global burden of heart failure: a comprehensive and updated review of epidemiology. Cardiovasc Res.

[9] Hancock HC, Close H, Fuat A, Murphy JJ, Hungin AP, Mason JM (2014). Barriers to accurate diagnosis and effective management of heart failure have not changed in the past 10 years: a qualitative study and national survey. BMJ Open.

[10] Smeets M, Zervas S, Leben H (2019). General practitioners' perceptions about their role in current and future heart failure care: an exploratory qualitative study. BMC Health Serv Res.

[11] Jaarsma T, Hill L, Bayes-Genis A (2021). Self-care of heart failure patients: practical management recommendations from the Heart Failure Association of the European Society of Cardiology. Eur J Heart Fail.

[12] Ravingerová T, Kindernay L, Barteková M (2020). The molecular mechanisms of iron metabolism and its role in cardiac dysfunction and cardioprotection. Int J Mol Sci.

[13] Zhang F, Lin JJ, Tian HN, Wang J (2024). Effect of exercise on improving myocardial mitochondrial function in decreasing diabetic cardiomyopathy. Exp Physiol.

[14] Li A, Gao M, Jiang W, Qin Y, Gong G (2020). Mitochondrial dynamics in adult cardiomyocytes and heart diseases. Front Cell Dev Biol.

[15] Pollock JD, Makaryus AN (2024). Physiology, Cardiac Cycle. https://www.ncbi.nlm.nih.gov/books/NBK459327/.

[16] Jeon YH, He M, Austin J, Shin H, Pfleger J, Abdellatif M (2021). Adiponectin enhances the bioenergetics of cardiac myocytes via an AMPK- and succinate dehydrogenase-dependent mechanism. Cell Signal.

[17] Guo Q, Zhang Y, Zhang S (2020). Genome-wide translational reprogramming of genes important for myocyte functions in overload-induced heart failure. Biochim Biophys Acta Mol Basis Dis.

[18] Koller A, Laughlin MH, Cenko E (2022). Functional and structural adaptations of the coronary macro- and microvasculature to regular aerobic exercise by activation of physiological, cellular, and molecular mechanisms: ESC Working Group on Coronary Pathophysiology and Microcirculation position paper. Cardiovasc Res.

[19] Amin MN, Siddiqui SA, Ibrahim M, Hakim ML, Ahammed MS, Kabir A, Sultana F (2020). Inflammatory cytokines in the pathogenesis of cardiovascular disease and cancer. SAGE Open Med.

[20] Azevedo PS, Polegato BF, Minicucci MF, Paiva SA, Zornoff LA (2016). Cardiac remodeling: concepts, clinical impact, pathophysiological mechanisms and pharmacologic treatment. Arq Bras Cardiol.

[21] Pugliese NR, Pellicori P, Filidei F (2023). Inflammatory pathways in heart failure with preserved left ventricular ejection fraction: implications for future interventions. Cardiovasc Res.

[22] Ghafourian K, Shapiro JS, Goodman L, Ardehali H (2020). Iron and heart failure: diagnosis, therapies, and future directions. JACC Basic Transl Sci.

[23] Samson R, Ramachandran R, Le Jemtel TH (2014). Systolic heart failure: knowledge gaps, misconceptions, and future directions. Ochsner J.

[24] Obokata M, Reddy YN, Borlaug BA (2020). Diastolic dysfunction and heart failure with preserved ejection fraction: understanding mechanisms by using noninvasive methods. JACC Cardiovasc Imaging.

[25] Mauro C, Chianese S, Cocchia R (2023). Acute heart failure: diagnostic-therapeutic pathways and preventive strategies—a real-world clinician’s guide. J Clin Med.

[26] Mado H, Szczurek W, Gąsior M, Szyguła-Jurkiewicz B (2021). Adiponectin in heart failure. Future Cardiol.

[27] Triposkiadis F, Sarafidis P, Briasoulis A, Magouliotis DE, Athanasiou T, Skoularigis J, Xanthopoulos A (2023). Hypertensive heart failure. J Clin Med.

[28] Tackling G, Borhade MB (2024). Hypertensive Heart Disease. http:////www.ncbi.nlm.nih.gov/books/NBK539800/.

[29] Rosano GM, Vitale C, Seferovic P (2017). Heart failure in patients with diabetes mellitus. Card Fail Rev.

[30] Watson RD, Gibbs CR, Lip GY (2000). ABC of heart failure. Clinical features and complications. BMJ.

[31] Pitt B (1992). The role of beta-adrenergic blocking agents in preventing sudden cardiac death. Circulation.

[32] Kennel PJ, Mancini DM, Schulze PC (2015). Skeletal muscle changes in chronic cardiac disease and failure. Compr Physiol.

[33] Anker SD, Ponikowski P, Varney S (1997). Wasting as independent risk factor for mortality in chronic heart failure. Lancet.

[34] Novack ML, Zubair M (2024). Natriuretic Peptide B Type Test. StatPearls.

[35] Arnold SV (2023). Assessment of the patient with heart failure symptoms and risk factors: a guide for the non-cardiologist. Diabetes Obes Metab.

[36] Biasucci LM, Maino A, Grimaldi MC, Cappannoli L, Aspromonte N (2021). Novel biomarkers in heart failure: new insight in pathophysiology and clinical perspective. J Clin Med.

[37] Wang YC, Yu CC, Chiu FC, Tsai CT, Lai LP, Hwang JJ, Lin JL (2013). Soluble ST2 as a biomarker for detecting stable heart failure with a normal ejection fraction in hypertensive patients. J Card Fail.

[38] Aimo A, Vergaro G, Ripoli A (2017). Meta-analysis of soluble suppression of tumorigenicity-2 and prognosis in acute heart failure. JACC Heart Fail.

[39] Boisot S, Beede J, Isakson S (2008). Serial sampling of ST2 predicts 90-day mortality following destabilized heart failure. J Card Fail.

[40] Manzano-Fernández S, Januzzi JL, Pastor-Pérez FJ (2012). Serial monitoring of soluble interleukin family member ST2 in patients with acutely decompensated heart failure. Cardiology.

[41] Emdin M, Aimo A, Vergaro G (2018). sST2 predicts outcome in chronic heart failure beyond NT-proBNP and high-sensitivity troponin T. J Am Coll Cardiol.

[42] Zilinski JL, Shah RV, Gaggin HK, Gantzer ML, Wang TJ, Januzzi JL (2012). Measurement of multiple biomarkers in advanced stage heart failure patients treated with pulmonary artery catheter guided therapy. Crit Care.

[43] Liquori ME, Christenson RH, Collinson PO, Defilippi CR (2014). Cardiac biomarkers in heart failure. Clin Biochem.

[44] Dumic J, Dabelic S, Flögel M (2006). Galectin-3: an open-ended story. Biochim Biophys Acta.

[45] Henderson NC, Mackinnon AC, Farnworth SL (2008). Galectin-3 expression and secretion links macrophages to the promotion of renal fibrosis. Am J Pathol.

[46] Song X, Qian X, Shen M (2015). Protein kinase C promotes cardiac fibrosis and heart failure by modulating galectin-3 expression. Biochim Biophys Acta.

[47] Suthahar N, Meijers WC, Silljé HH, Ho JE, Liu FT, de Boer RA (2018). Galectin-3 activation and inhibition in heart failure and cardiovascular disease: an update. Theranostics.

[48] Ueland T, Aukrust P, Broch K, Aakhus S, Skårdal R, Muntendam P, Gullestad L (2011). Galectin-3 in heart failure: high levels are associated with all-cause mortality. Int J Cardiol.

[49] Ho JE, Liu C, Lyass A (2012). Galectin-3, a marker of cardiac fibrosis, predicts incident heart failure in the community. J Am Coll Cardiol.

[50] Ahmad T, Felker GM (2012). Galectin-3 in heart failure: more answers or more questions?. J Am Heart Assoc.

[51] van Kimmenade RR, Januzzi JL Jr, Ellinor PT (2006). Utility of amino-terminal pro-brain natriuretic peptide, galectin-3, and apelin for the evaluation of patients with acute heart failure. J Am Coll Cardiol.

[52] Daniels LB, Clopton P, Laughlin GA, Maisel AS, Barrett-Connor E (2014). Galectin-3 is independently associated with cardiovascular mortality in community-dwelling older adults without known cardiovascular disease: the Rancho Bernardo Study. Am Heart J.

[53] de Boer RA, van Veldhuisen DJ, Gansevoort RT (2012). The fibrosis marker galectin-3 and outcome in the general population. J Intern Med.

[54] Darden D, Nishimura M, Sharim J, Maisel A (2019). An update on the use and discovery of prognostic biomarkers in acute decompensated heart failure. Expert Rev Mol Diagn.

[55] Yancy CW, Jessup M, Bozkurt B (2017). 2017 ACC/AHA/HFSA focused update of the 2013 ACCF/AHA guideline for the management of heart failure: a report of the American College of Cardiology/American Heart Association Task Force on Clinical Practice Guidelines and the Heart Failure Society of America. Circulation.

[56] Lok DJ, Van Der Meer P, de la Porte PW, Lipsic E, Van Wijngaarden J, Hillege HL, van Veldhuisen DJ (2010). Prognostic value of galectin-3, a novel marker of fibrosis, in patients with chronic heart failure: data from the DEAL-HF study. Clin Res Cardiol.

[57] Tang WH, Shrestha K, Shao Z, Borowski AG, Troughton RW, Thomas JD, Klein AL (2011). Usefulness of plasma galectin-3 levels in systolic heart failure to predict renal insufficiency and survival. Am J Cardiol.

[58] López B, González A, Ravassa S (2015). Circulating biomarkers of myocardial fibrosis: the need for a reappraisal. J Am Coll Cardiol.

[59] Gyöngyösi M, Winkler J, Ramos I (2017). Myocardial fibrosis: biomedical research from bench to bedside. Eur J Heart Fail.

[60] González A, Schelbert EB, Díez J, Butler J (2018). Myocardial interstitial fibrosis in heart failure: biological and translational perspectives. J Am Coll Cardiol.

[61] Klappacher G, Franzen P, Haab D (1995). Measuring extracellular matrix turnover in the serum of patients with idiopathic or ischemic dilated cardiomyopathy and impact on diagnosis and prognosis. Am J Cardiol.

[62] Weber KT, Pick R, Jalil JE, Janicki JS, Carroll EP (1989). Patterns of myocardial fibrosis. J Mol Cell Cardiol.

[63] Löfsjögård J, Kahan T, Díez J (2014). Biomarkers of collagen type I metabolism are related to B-type natriuretic peptide, left ventricular size, and diastolic function in heart failure. J Cardiovasc Med (Hagerstown).

[64] Krum H, Elsik M, Schneider HG (2011). Relation of peripheral collagen markers to death and hospitalization in patients with heart failure and preserved ejection fraction: results of the I-PRESERVE collagen substudy. Circ Heart Fail.

[65] Flevari P, Theodorakis G, Leftheriotis D (2012). Serum markers of deranged myocardial collagen turnover: their relation to malignant ventricular arrhythmias in cardioverter-defibrillator recipients with heart failure. Am Heart J.

[66] Ramseyer VD, Cabral PD, Garvin JL (2011). Role of endothelin in thick ascending limb sodium chloride transport. Contrib Nephrol.

[67] López B, Ravassa S, González A (2016). Myocardial collagen cross-linking is associated with heart failure hospitalization in patients with hypertensive heart failure. J Am Coll Cardiol.

[68] Masson S, Latini R, Anand IS (2006). The prognostic value of big endothelin-1 in more than 2,300 patients with heart failure enrolled in the Valsartan Heart Failure Trial (Val-HeFT). J Card Fail.

[69] Zymliński R, Sierpiński R, Metra M (2020). Elevated plasma endothelin-1 is related to low natriuresis, clinical signs of congestion, and poor outcome in acute heart failure. ESC Heart Fail.

[70] Askari AT, Brennan ML, Zhou X (2003). Myeloperoxidase and plasminogen activator inhibitor 1 play a central role in ventricular remodeling after myocardial infarction. J Exp Med.

[71] Tang WH, Brennan ML, Philip K, Tong W, Mann S, Van Lente F, Hazen SL (2006). Plasma myeloperoxidase levels in patients with chronic heart failure. Am J Cardiol.

[72] Tang WH, Katz R, Brennan ML, Aviles RJ, Tracy RP, Psaty BM, Hazen SL (2009). Usefulness of myeloperoxidase levels in healthy elderly subjects to predict risk of developing heart failure. Am J Cardiol.

[73] Chaikijurajai T, Tang WH (2020). Reappraisal of inflammatory biomarkers in heart failure. Curr Heart Fail Rep.

[74] Tang WH, Tong W, Troughton RW (2007). Prognostic value and echocardiographic determinants of plasma myeloperoxidase levels in chronic heart failure. J Am Coll Cardiol.

[75] Sayed-Ahmed MM, Khattab MM, Gad MZ, Osman AM (2001). Increased plasma endothelin-1 and cardiac nitric oxide during doxorubicin-induced cardiomyopathy. Pharmacol Toxicol.

[76] Ky B, Putt M, Sawaya H (2014). Early increases in multiple biomarkers predict subsequent cardiotoxicity in patients with breast cancer treated with doxorubicin, taxanes, and trastuzumab. J Am Coll Cardiol.

[77] George M, Jena A, Srivatsan V, Muthukumar R, Dhandapani VE (2016). GDF 15 - a novel biomarker in the offing for heart failure. Curr Cardiol Rev.

[78] Manhenke C, Ørn S, von Haehling S (2013). Clustering of 37 circulating biomarkers by exploratory factor analysis in patients following complicated acute myocardial infarction. Int J Cardiol.

[79] Eggers KM, Kempf T, Wallentin L, Wollert KC, Lind L (2013). Change in growth differentiation factor 15 concentrations over time independently predicts mortality in community-dwelling elderly individuals. Clin Chem.

[80] Dispenzieri A, Kyle R, Merlini G (2009). International Myeloma Working Group guidelines for serum-free light chain analysis in multiple myeloma and related disorders. Leukemia.

[81] Esparvarinha M, Nickho H, Mohammadi H, Aghebati-Maleki L, Abdolalizadeh J, Majidi J (2017). The role of free kappa and lambda light chains in the pathogenesis and treatment of inflammatory diseases. Biomed Pharmacother.

[82] Bellary S, Faint JM, Assi LK, Hutchison CA, Harding SJ, Raymond NT, Barnett AH (2014). Elevated serum free light chains predict cardiovascular events in type 2 diabetes. Diabetes Care.

[83] Shantsila E, Tapp LD, Lip GY (2015). Free light chains in patients with acute coronary syndromes: relationships to inflammation and renal function. Int J Cardiol.

[84] Shantsila E, Wrigley B, Lip GY (2014). Free light chains in patients with acute heart failure secondary to atherosclerotic coronary artery disease. Am J Cardiol.

[85] Burmeister A, Assi LK, Ferro CJ (2014). The relationship between high-sensitivity CRP and polyclonal free light chains as markers of inflammation in chronic disease. Int J Lab Hematol.

[86] Nakamura R, Kato J, Kitamura K (2004). Adrenomedullin administration immediately after myocardial infarction ameliorates progression of heart failure in rats. Circulation.

[87] Voors AA, Kremer D, Geven C (2019). Adrenomedullin in heart failure: pathophysiology and therapeutic application. Eur J Heart Fail.

[88] Pousset F, Masson F, Chavirovskaia O (2000). Plasma adrenomedullin, a new independent predictor of prognosis in patients with chronic heart failure. Eur Heart J.

[89] Stoiser B, Mörtl D, Hülsmann M (2006). Copeptin, a fragment of the vasopressin precursor, as a novel predictor of outcome in heart failure. Eur J Clin Invest.

[90] Zhong Y, Wang R, Yan L, Lin M, Liu X, You T (2017). Copeptin in heart failure: review and meta-analysis. Clin Chim Acta.

[91] Wang KC, Chang HY (2011). Molecular mechanisms of long noncoding RNAs. Mol Cell.

[92] Saxena A, Carninci P (2011). Long non-coding RNA modifies chromatin: epigenetic silencing by long non-coding RNAs. Bioessays.

[93] Kung JT, Colognori D, Lee JT (2013). Long noncoding RNAs: past, present, and future. Genetics.

[94] Zhang Z, Gao W, Long QQ (2017). Increased plasma levels of lncRNA H19 and LIPCAR are associated with increased risk of coronary artery disease in a Chinese population. Sci Rep.

[95] Luo H, Wang J, Liu D (2019). The lncRNA H19/miR-675 axis regulates myocardial ischemic and reperfusion injury by targeting PPARα. Mol Immunol.

[96] Micheletti R, Plaisance I, Abraham BJ (2017). The long noncoding RNA Wisper controls cardiac fibrosis and remodeling. Sci Transl Med.

[97] Wang Z, Zhang XJ, Ji YX (2016). The long noncoding RNA Chaer defines an epigenetic checkpoint in cardiac hypertrophy. Nat Med.

[98] Viereck J, Kumarswamy R, Foinquinos A (2016). Long noncoding RNA Chast promotes cardiac remodeling. Sci Transl Med.

[99] McPherson R, Pertsemlidis A, Kavaslar N (2007). A common allele on chromosome 9 associated with coronary heart disease. Science.

[100] Dai W, Lee D (2019). Interfering with long chain noncoding RNA ANRIL expression reduces heart failure in rats with diabetes by inhibiting myocardial oxidative stress. J Cell Biochem.

[101] Zhang M, Gu H, Xu W, Zhou X (2016). Down-regulation of lncRNA MALAT1 reduces cardiomyocyte apoptosis and improves left ventricular function in diabetic rats. Int J Cardiol.

[102] de Gonzalo-Calvo D, Kenneweg F, Bang C (2016). Circulating long-non coding RNAs as biomarkers of left ventricular diastolic function and remodelling in patients with well-controlled type 2 diabetes. Sci Rep.

[103] Boeckel JN, Perret MF, Glaser SF (2019). Identification and regulation of the long non-coding RNA Heat2 in heart failure. J Mol Cell Cardiol.

[104] Boros J, Arnoult N, Stroobant V, Collet JF, Decottignies A (2014). Polycomb repressive complex 2 and H3K27me3 cooperate with H3K9 methylation to maintain heterochromatin protein 1α at chromatin. Mol Cell Biol.

[105] Zhang J, Gao C, Meng M, Tang H (2016). Long noncoding RNA MHRT protects cardiomyocytes against H2O2-induced apoptosis. Biomol Ther (Seoul).

[106] Han P, Li W, Lin CH (2014). A long noncoding RNA protects the heart from pathological hypertrophy. Nature.

[107] Greco S, Zaccagnini G, Perfetti A (2016). Long noncoding RNA dysregulation in ischemic heart failure. J Transl Med.

[108] Ciampi Q, Villari B (2007). Role of echocardiography in diagnosis and risk stratification in heart failure with left ventricular systolic dysfunction. Cardiovasc Ultrasound.

[109] Carroll JD, Lang RM, Neumann AL, Borow KM, Rajfer SI (1986). The differential effects of positive inotropic and vasodilator therapy on diastolic properties in patients with congestive cardiomyopathy. Circulation.

[110] Milos RI, Bartha C, Röhrich S (2023). Imaging in patients with acute dyspnea when cardiac or pulmonary origin is suspected. BJR Open.

[111] Agasthi P, Chao CJ, Siegel RJ (2020). Comparison of echocardiographic parameters with cardiac magnetic resonance imaging in the assessment of right ventricular function. Echocardiography.

[112] Situ Y, Birch SC, Moreyra C, Holloway CJ (2020). Cardiovascular magnetic resonance imaging for structural heart disease. Cardiovasc Diagn Ther.

[113] Guo R, Weingärtner S, Šiurytė P (2022). Emerging techniques in cardiac magnetic resonance imaging. J Magn Reson Imaging.

[114] Giordano C, Francone M, Cundari G, Pisano A, d'Amati G (2022). Myocardial fibrosis: morphologic patterns and role of imaging in diagnosis and prognostication. Cardiovasc Pathol.

[115] Huang S, Xu HY, Diao KY (2020). Left ventricular global function index by magnetic resonance imaging - a novel marker for differentiating cardiac amyloidosis from hypertrophic cardiomyopathy. Sci Rep.

[116] Ibrahim EH, Dennison J, Frank L, Stojanovska J (2021). Diastolic cardiac function by MRI—imaging capabilities and clinical applications. Tomography.

[117] Kabasawa H (2022). MR imaging in the 21st century: technical innovation over the first two decades. Magn Reson Med Sci.

[118] Burrage MK, Ferreira VM (2020). Cardiovascular magnetic resonance for the differentiation of left ventricular hypertrophy. Curr Heart Fail Rep.

[119] Han D, Miller RJ, Otaki Y (2021). Diagnostic accuracy of cardiovascular magnetic resonance for cardiac transplant rejection: a meta-analysis. JACC Cardiovasc Imaging.

[120] Hindieh W, Weissler-Snir A, Hammer H, Adler A, Rakowski H, Chan RH (2017). Discrepant measurements of maximal left ventricular wall thickness between cardiac magnetic resonance imaging and echocardiography in patients with hypertrophic cardiomyopathy. Circ Cardiovasc Imaging.

[121] Grajewski KG, Stojanovska J, Ibrahim EH, Sayyouh M, Attili A (2020). Left ventricular hypertrophy: evaluation with cardiac MRI. Curr Probl Diagn Radiol.

[122] Aquaro GD, Monastero S, Todiere G (2023). Diagnostic role of native T1 mapping compared to conventional magnetic resonance techniques in cardiac disease in a real-life cohort. Diagnostics (Basel).

[123] Poindron V, Chatelus E, Canuet M (2020). T1 mapping cardiac magnetic resonance imaging frequently detects subclinical diffuse myocardial fibrosis in systemic sclerosis patients. Semin Arthritis Rheum.

[124] Hassan S, Barrett CJ, Crossman DJ (2020). Imaging tools for assessment of myocardial fibrosis in humans: the need for greater detail. Biophys Rev.

[125] Li R, Edalati M, Muccigrosso D, Lau JM, Laforest R, Woodard PK, Zheng J (2023). A simplified method to correct saturation of arterial input function for cardiac magnetic resonance first-pass perfusion imaging: validation with simultaneously acquired PET. J Cardiovasc Magn Reson.

[126] Assadi H, Jones R, Swift AJ, Al-Mohammad A, Garg P (2021). Cardiac MRI for the prognostication of heart failure with preserved ejection fraction: a systematic review and meta-analysis. Magn Reson Imaging.

[127] El-Sayed ZH, El-Samei MMA, Mohamed GI, Mohamed MM (2022). Role of stress echocardiography in assessment of myocardial viability and contractile reserve. NeuroQuantology.

[128] Almeida AG, Carpenter JP, Cameli M (2021). Multimodality imaging of myocardial viability: an expert consensus document from the European Association of Cardiovascular Imaging (EACVI). Eur Heart J Cardiovasc Imaging.

[129] Jada L, Holtackers RJ, Martens B (2024). Quantification of myocardial scar of different etiology using dark- and bright-blood late gadolinium enhancement cardiovascular magnetic resonance. Sci Rep.

[130] Behera DR, Kumar VKA, Namboodiri KKN (2020). Prognostic value of late gadolinium enhancement in cardiac MRI of non-ischemic dilated cardiomyopathy patients. Indian Heart J.

[131] Nakamori S, Dohi K (2022). Myocardial tissue imaging with cardiovascular magnetic resonance. J Cardiol.

[132] Bakouri M, Alassaf A, Alshareef K, Abdelsalam S, Ismail HF, Ganoun A, Alomari AH (2022). An optimal H-infinity controller for left ventricular assist devices based on a Starling-like controller: a simulation study. Mathematics.

[133] Ford TJ, Ong P, Sechtem U (2020). Assessment of vascular dysfunction in patients without obstructive coronary artery disease: why, how, and when. JACC Cardiovasc Interv.

[134] Brener MI, Masoumi A, Ng VG (2022). Invasive right ventricular pressure-volume analysis: basic principles, clinical applications, and practical recommendations. Circ Heart Fail.

[135] Grinstein J, Houston BA, Nguyen AB (2023). Standardization of the right heart catheterization and the emerging role of advanced hemodynamics in heart failure. J Card Fail.

[136] Khandhar SJ, Mehta M, Cilia L, Palevsky H, Matthai W, Rivera-Lebron B, Toma C (2020). Invasive hemodynamic assessment of patients with submassive pulmonary embolism. Catheter Cardiovasc Interv.

[137] Saugel B, Kouz K, Scheeren TW, Greiwe G, Hoppe P, Romagnoli S, de Backer D (2021). Cardiac output estimation using pulse wave analysis—physiology, algorithms, and technologies: a narrative review. Br J Anaesth.

[138] Verbrugge FH, Guazzi M, Testani JM, Borlaug BA (2020). Altered hemodynamics and end-organ damage in heart failure: Impact on the lung and kidney. Circulation.

[139] Hsu S, Fang JC, Borlaug BA (2022). Hemodynamics for the heart failure clinician: a state-of-the-art review. J Card Fail.

[140] Narula J, Chandrashekhar Y, Ahmadi A (2021). SCCT 2021 expert consensus document on coronary computed tomographic angiography: a report of the Society of Cardiovascular Computed Tomography. J Cardiovasc Comput Tomogr.

[141] Lell MM, Kachelrieß M (2020). Recent and upcoming technological developments in computed tomography: high speed, low dose, deep learning, multienergy. Invest Radiol.

[142] Mann A, Williams J (2020). Considerations for stress testing performed in conjunction with myocardial perfusion imaging. J Nucl Med Technol.

[143] Yang H, Faust E, Gao E (2022). Evaluating the use of pharmacological stress agents during single-photon emission computed tomography myocardial perfusion imaging tests after inadequate exercise stress test. J Nucl Cardiol.

[144] Elkholy KO, Hegazy O, Okunade A, Aktas S, Ajibawo T (2021). Regadenoson stress testing: a comprehensive review with a focused update. Cureus.

[145] Čelutkienė J, Pudil R, López-Fernández T (2020). Role of cardiovascular imaging in cancer patients receiving cardiotoxic therapies: a position statement on behalf of the Heart Failure Association (HFA), the European Association of Cardiovascular Imaging (EACVI) and the Cardio-Oncology Council of the European Society of Cardiology (ESC). Eur J Heart Fail.

[146] Meng H, Ruan J, Yan Z, Chen Y, Liu J, Li X, Meng F (2022). New progress in early diagnosis of atherosclerosis. Int J Mol Sci.

[147] Friedman MM (1997). Older adults' symptoms and their duration before hospitalization for heart failure. Heart Lung.

[148] Jurgens CY (2006). Somatic awareness, uncertainty, and delay in care-seeking in acute heart failure. Res Nurs Health.

[149] Lenz ER, Pugh LC, Milligan RA, Gift A, Suppe F (1997). The middle-range theory of unpleasant symptoms: an update. ANS Adv Nurs Sci.

[150] Lenz ER, Suppe F, Gift AG, Pugh LC, Milligan RA (1995). Collaborative development of middle-range nursing theories: toward a theory of unpleasant symptoms. ANS Adv Nurs Sci.

[151] Yu DS, Chan HY, Leung DY, Hui E, Sit JW (2016). Symptom clusters and quality of life among patients with advanced heart failure. J Geriatr Cardiol.

[152] Moser DK, Riegel B (2008). Cardiac Nursing: A Companion to Braunwald's Heart Disease. https://search.worldcat.org/title/181902809.

[153] Severino P, D'Amato A, Prosperi S (2022). Do the current guidelines for heart failure diagnosis and treatment fit with clinical complexity?. J Clin Med.

[154] Severino P, D'Amato A, Prosperi S (2023). Heart failure pharmacological management: gaps and current perspectives. J Clin Med.

[155] Smiseth OA, Morris DA, Cardim N (2022). Multimodality imaging in patients with heart failure and preserved ejection fraction: an expert consensus document of the European Association of Cardiovascular Imaging. Eur Heart J Cardiovasc Imaging.

[156] Pergola V, Cameli M, Mattesi G (2023). Multimodality imaging in advanced heart failure for diagnosis, management and follow-up: a comprehensive review. J Clin Med.

[157] D'Andrea A, Ilardi F, Palermi S (2023). Multimodality imaging in decompensated heart failure. Eur Heart J Suppl.

[158] Robinson S, Ring L, Oxborough D (2024). The assessment of left ventricular diastolic function: guidance and recommendations from the British Society of Echocardiography. Echo Res Pract.

[159] Hamet P, Tremblay J (2017). Artificial intelligence in medicine. Metabolism.

[160] Rajkomar A, Dean J, Kohane I (2019). Machine learning in medicine. N Engl J Med.

[161] Barry T, Farina JM, Chao CJ (2023). The role of artificial intelligence in echocardiography. J Imaging.

[162] Chen R, Lu A, Wang J (2019). Using machine learning to predict one-year cardiovascular events in patients with severe dilated cardiomyopathy. Eur J Radiol.

[163] Kusunose K, Haga A, Yamaguchi N (2020). Deep learning for assessment of left ventricular ejection fraction from echocardiographic images. J Am Soc Echocardiogr.

[164] Yamaguchi N, Kosaka Y, Haga A, Sata M, Kusunose K (2023). Artificial intelligence-assisted interpretation of systolic function by echocardiogram. Open Heart.

[165] Attia ZI, Kapa S, Lopez-Jimenez F (2019). Screening for cardiac contractile dysfunction using an artificial intelligence-enabled electrocardiogram. Nat Med.

[166] Kwon JM, Kim KH, Jeon KH (2019). Development and validation of deep-learning algorithm for electrocardiography-based heart failure identification. Korean Circ J.

[167] Kwon JM, Kim KH, Eisen HJ (2021). Artificial intelligence assessment for early detection of heart failure with preserved ejection fraction based on electrocardiographic features. Eur Heart J Digit Health.

[168] Sutton RT, Pincock D, Baumgart DC, Sadowski DC, Fedorak RN, Kroeker KI (2020). An overview of clinical decision support systems: benefits, risks, and strategies for success. NPJ Digit Med.

[169] Choi DJ, Park JJ, Ali T, Lee S (2020). Artificial intelligence for the diagnosis of heart failure. NPJ Digit Med.

[170] Kristiansen TB, Kristensen K, Uffelmann J, Brandslund I (2022). Erroneous data: the Achilles' heel of AI and personalized medicine. Front Digit Health.

